# A Systemic Review of Resistance Mechanisms and Ongoing Clinical Trials in ALK-Rearranged Non-Small Cell Lung Cancer

**DOI:** 10.3389/fonc.2014.00174

**Published:** 2014-07-21

**Authors:** Khashayar Esfahani, Jason Scott Agulnik, Victor Cohen

**Affiliations:** ^1^Department of Oncology, Segal Cancer Center, Sir Mortimer B. Davis Jewish General Hospital, Montreal, QC, Canada; ^2^Division of Pulmonary Diseases, Department of Oncology, Peter Brojde Cancer Center, Montreal, QC, Canada

**Keywords:** non-small cell lung cancer, driver mutations, anaplastic lymphoma kinase, crizotinib, ALK-inhibitors, clinical trials as topic

## Abstract

The identification of oncogenic driver mutations in non-small cell lung cancer (NSCLC) has led to a paradigm shift and the development of specific molecular treatments. Tumors harboring a rearranged EML4–ALK fusion oncogene are highly sensitive to therapy with ALK-targeted inhibitors. Crizotinib is the first approved treatment for advanced lung tumors containing this genetic abnormality. In this mini review, we discuss the existing data on crizotinib as well as ongoing trials involving this medication. A brief overview of the known resistance mechanisms to crizotinib will also be presented followed by a summary of the ongoing trials involving next-generation ALK-inhibitors or other targeted therapies in patients with ALK+ NSCLC.

## Introduction

Treatment for non-small cell lung cancer (NSCLC) has historically consisted of cytotoxic chemotherapy. Recent advances in molecular biology had led to the discovery of oncogenic driver mutations with subsequent development of oral agents that target these molecular pathways. In NSCLC, the main two driver mutations, with FDA approved targeted therapies, consist of echinoderm microtubule protein like-4/anaplastic lymphoma kinase (EML-4/ALK) translocations and epidermal growth factor receptor (EGFR) mutations. Crizotinib is the first FDA approved treatment for patients with ALK+ NSCLC. To this date, only the final data from one phase III randomized trial has been published, evaluating the use of crizotinib as a second-line therapy (1). Multiple phase III randomized trials are in progress to assess the efficacy of crizotinib as first-line chemotherapy. Eventually, most patients on treatment with crizotinib develop resistance to this drug within 1 year of treatment. Most clinical trials in progress in the ALK+ patient population involve “new-generation ALK-inhibitors,” or crizotinib in combination with novel drugs to bypass known resistance mechanisms.

The scope of this review article is twofold. First, the existing data on crizotinib will be presented as well ongoing trials involving this medication. Second, a brief overview of the known resistance mechanisms to crizotinib will be presented followed by a summary of the ongoing trials involving newer generation ALK-inhibitors or other targeted therapies in patients with ALK+ NSCLC.

## ALK Fusion Gene and Its Target Crizotinib

**Figure 1:**
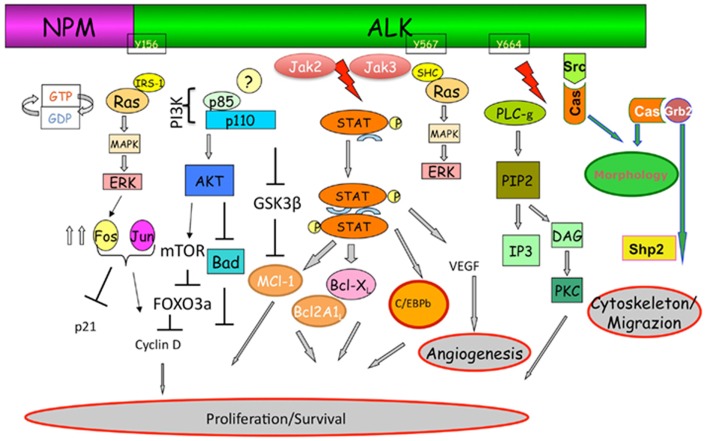
**The ALK signaling pathway with its cross-talk with other pathways involved in the resistance to ALK-inhibitors**. Adapted from Tabbo et al. (47).

Crizotinib is an orally active inhibitor of multiple tyrosine kinase inhibitors (TKIs), including ALK, c-Met, hepatocyte growth factor receptor (HGFR), and c-ros oncogene 1 (ROS1) (Figure 1) (2). In 2007, the ALK gene rearrangement in which the 5′ end of EML4 gene is fused to the 3′ end of ALK was first identified by Soda et al. in NSCLC cell lines (3). The fusion protein resulting from this translocation has constitutive kinase activity, leading to downstream activation of multiple diverse signaling cascades involved in cell proliferation and carcinogenesis. Currently, multiple EML4–ALK fusion combinations have been identified (4). All these fusion proteins have a similar ALK kinase domain, but differ in the EML4 breakpoint. Pre-clinical data from *in vitro* studies suggested different crizotinib sensitivity for each variant of the EML4–ALK fusion protein (5). However, a subgroup analysis from the phase I trial of crizotinib failed to demonstrate such correlation between variant fusion proteins and clinical response to therapy (6). In addition, fusions of ALK with other partners including TRK-fused gene TFG and KIF5B have also been described in lung cancer patients, but appear to be much less common than EML4-ALK (7).

## Clinical Trials Involving Crizotinib

In 2013, Shaw et al. published the first phase III randomized trial involving crizotinib in the second-line setting (1). Patients with locally advanced or metastatic ALK+ NSCLC were randomly assigned to receive oral treatment with crizotinib (250 mg) twice daily or intravenous chemotherapy with either pemetrexed or docetaxel. The median progression-free survival was 7.7 months in the crizotinib group and 3.0 months in the chemotherapy group. An interim analysis of overall survival showed no significant improvement with crizotinib as compared with chemotherapy. This analysis was nevertheless immature with a total of 96 deaths (40% of the required events) and censoring of over 70% of patients in either treatment arm. In addition, the analysis was likely confounded by the high crossover rate of patients in the chemotherapy group, with nearly 90% of patients on the chemotherapy arm crossing over to the other arm upon disease progression. The response rates were 65% with crizotinib, as compared with 20% with chemotherapy. Common adverse events associated with crizotinib were visual disorder, gastrointestinal side effects, and elevated liver aminotransferase levels. Given the positive response rates with crizotinib, multiple phase III trials are currently in progress to address the efficacy of crizotinib as first-line therapy. Details of these trials, including patient population and their respective primary endpoints, are summarized in Table S1 in Supplementary Material.

One challenging clinical problem remains the treatment of ALK+ NSCLC patients with brain metastasis. These patients suffer from an adverse impact on quality of life and survival. Although it has been shown that crizotinib is effective for brain metastasis, it is penetration into cerebrospinal fluid (CSF) has been demonstrated to be very poor (8). Costa et al. measured the CSF-to-plasma ratio of crizotinib being only at 0.0026 (9). Past experience with erlotinib and gefitinib in patients with EGFR-mutated lung cancer has uncovered similar challenges: despite good systemic control of disease, a subset of patients would progress in the CNS, without any new acquired resistance mechanism, owing to the poor penetration of these TKIs in the CSF. Although pulse EGFR–TKIs doses have been used in this setting, there is limited data to support its use with crizotinib (10). Newer generation of ALK-inhibitors with better CSF penetration are currently under study.

## Resistance Mechanisms of Crizotinib

In order to understand the rationale behind the majority of ongoing clinical trials involving ALK+ NSCLC patients, it is important to survey the currently known mechanism of resistance to crizotinib. ALK-dependant resistance mechanism occurs upon mutations in the tyrosine kinase (TK) domain, and activation of alternative signaling pathways. Alternatively, true ALK-independent resistance may arise through the outgrowth of clones that do not harbor an ALK gene fusion and contain a separate activated oncogene (11). Given that multiple resistance mechanisms are occasionally found within the same biopsy specimen, as well as different resistance mechanisms may be found in separate tumor deposits within the same patient, it is important to consider re-biopsy of the tumor upon progression on treatment, whenever technically feasible, to correctly identify the resistance mechanism accounting for progression of disease (12).

### Mutations in target tyrosine kinases

Past experience with the use of TKIs in chronic myelogenous leukemia as well as EGFR-mutated lung cancer teaches us that most common mechanisms of resistance to this class of medications are secondary mutations in the TK domains (13). This also holds true for crizotinib, given that mutations in the TK domains of the different targets of crizotinib is currently the best studied and most prevalent form of resistance to this drug, accounting for up to 25% of all cases resistant to ALK therapy (14).

The first major “gatekeeper” mutation identified in the TK domain of EML4–ALK involves the substitution of leucine for a methionine at position 1196 (L1196M) of the kinase domain of ALK, thus creating a mutant bulky amino-acid side chain in the ATP-binding pocket of the receptor, ultimately interfering with the binding of crizotinib to its receptor (15). This is analogous to the EGFR T790M mutation in the ATP biding pocket of the EGFR, which is the most common mechanism of resistance in EGFR-mutated lung cancer (12).

Tissue analysis of harvested tumor cells from patients resistant to crizotinib has demonstrated other non-gatekeeper secondary mutations in the ALK TK receptor. ALK secondary mutations in NSCLC are distributed throughout the kinase domain, including the solvent front (G1202R, S1206Y), ATP-binding pocket (G1269A), and N-terminal to the C-helix (1151Tins, F1174L, L1152R, and C1156Y) (11, 15–20). The prevalence and clinical significance of these secondary mutations remains to be elucidated. Of interest, a separate secondary ALK mutation, F1174L, has also been identified in inflammatory myofibroblastic tumors (21).

Crizotinib is an inhibitor of multiple TKIs beside ALK, including ROS1 oncogene. A recent report of a G2032R mutation in the ROS1 TK domain leading to crizotinib resistance has also been identified (22). Although this mutation does not lie at the gatekeeper position, it confers resistance to ROS1 kinase inhibition through steric interference with drug binding.

### Activation of alternative pathways

Activation of alternative downstream signaling pathways, even in the setting of complete ALK receptor inhibition, is increasingly recognized as mechanisms of resistance to crizotinib. These include activation of the EGFR, heat shock protein 90 (HSP90), and the PI3K/AKT/mTOR pathways. The activation of these alternative pathways is present in up to 20% of patients (23).

Recent data from cell line experiments showed the activation of EGFR to be associated with ALK resistance (18). This was further corroborated with clinical analysis of tumor cells biopsied from patients resistant to ALK therapy (24). In most studies, the activation of EGFR occurred through increased phosphorylation and upregulation of EGFR ligands, such as amphiregulin, rather than being caused by mutations in the EGFR gene itself (18, 25). Although ALK mutations are usually mutually exclusive to other driver mutations such as EGFR, there have been reports of *de novo* mutation of the EGFR gene in patients and cell lines treated with crizotinib, accounting for resistance to this drug (11, 24, 26, 27).

The PI3K/AKT/mTOR pathway is an intracellular signaling pathway important in cell cycle regulation and apoptosis also implicated in resistance to ALK-targeted therapy. Recent ALK-resistant cell line analysis revealed that the activation of the mTOR pathways was associated with increased autophagy of the ALK receptor thus leading to decreased response to crizotinib treatment (28). The exact mechanism by which the ALK receptor induces activation of the mTOR pathway remains to be elucidated, but inhibition of AKT by phosphorylation seems to play a key factor. One study showed synergistic *in vitro* growth inhibitory activity of ALK inhibitor when combined with an mTOR inhibitor (29). The clinical significance of mTOR activation in NSCLC patients remains to be elucidated.

HSP90 is a highly abundant and ubiquitous molecular chaperone, which plays an essential role in many cellular processes including cell cycle control, hormone signaling, as well as protein folding, and degradation. The ALK receptor is one of the many client proteins of HSP90. HSP90 inhibition induced loss of EML4-ALK expression and depletion of multiple oncogenic signaling proteins in ALK-driven NSCLC cell lines (30). These results were further corroborated in murine models of NSCLC as well as anecdotal case reports of tissues derived from ALK therapy resistant patients (31, 32).

## Ongoing Clinical Trials Involving New-Generation ALK-Inhibitors and Combination Therapy

Most ongoing trials in ALK+ NSCLC patients involve newer generation ALK-inhibitors or combination therapy targeting currently known resistance mechanism to crizotinib. These include agents with activity against NSCLC with the L1196M gatekeeper mutation or the ROS1 mutation, as well as combination therapy targeting the EGFR and HSP90 proteins/pathways. A summary of these trials are presented in Table 1. Current evidence for some of these new-generation ALK-inhibitors are presented here.

**Table 1:** **Ongoing clinical trials involving novel ALK- and HSP90-inhibitors in NSCLC**.

**ONGOING CLINICAL TRIALS INVOLVING NOVEL ALK-INHIBITORS**						
Drug	Company	Activity against	Activity against	Ongoing trials	Study phase	Previous treatment
		other kinases	L1196M mutation			
LDK378 (ceritinib)	Novartis	IFG-1R	Yes	NCT01772797	Phase I	None
		c-MET		NCT02040870	Phase I/II	Crizotinib/chemotherapy
				NCT01685138	Phase II	0-3 lines of chemotherapy
				NCT01685060	Phase II	Crizotinib or 1-3 lines of chemotherapy
				NCT01947608	Phase II	Crizotinib
				NCT01964157	Phase II	1 line of chemotherapy
				NCT01828099	Phase III	None
				NCT01828112	Phase III	Crizotinib
CH5424802/RO5424802 (alectinib)	Roche/Chugai	ROS1	Yes	NCT01588028	Phase I	
				NCT01871805	Phase II	Crizotinib
				NCT01801111	Phase II	Crizotinib
				NCT02075840	Phase III	
AP26113	Ariad	EGFR	Unknown	NCT01449461	Phase I/II	Refractory to standard therapy
		ROS1		NCT02094573	Phase II	Crizotinib
ASP3026	Astellas	ROS1	Yes	NCT01401504	Phase I	Refractory to standard therapy
				NCT01284192	Phase I	Refractory to standard therapy
TSR-001	Tesaro	Unknown	Yes	NCT02048488	Phase I	None
PF-06463922	Pfizer	EGFR	Unknown	NCT01970865	Phase I/II	None
		ROS1	
X-396	Xcovery	Unknown	Yes	NCT01625234	Phase I	None

**ONGOING CLINICAL TRIALS INVOLVING HSP90 INHIBITORS**						

**Drug**	**Company**	**Ongoing trials**	**Study phase**	**Combination therapy**		**Previous treatment**

AUY922	Novartis	NCT01772797	Phase I	LDK378		None
		NCT01752400	Phase II			Crizotinib
		NCT01124864	Phase II			Two lines of chemotherapy
		NCT01922583	Phase II			One line of chemotherapy
STA-9090	Synta	NCT01579994	Phase I/II	Crizotinib		Standard chemotherapy
		NCT01562015	Phase II			Three lines of therapy
IPI-504	Infinity	NCT01228435	Phase II			Refractory to standard therapy
AT13387	Astex	NCT01712217	Phase I/II	Crizotinib		Crizotinib
DS-2248	Daiichi Sankyo	NCT01288430	Phase I			Crizotinib

### Ceritinib (LDK378)

Ceritinib (LDK378, Novartis) is a novel and potent selective TKI targeting ALK. Results from a recent phase I/II study of this drug in both crizotinib-naïve and crizotinib-resistant patients have been published (33). The maximum tolerated dose of ceritinib was 750 mg once daily. Dose-limiting toxic events included diarrhea, vomiting, dehydration, elevated aminotransferase levels, and hypophosphatemia. Among 114 patients with NSCLC who received at least 400 mg of Ceritinib per day, the overall response rate was 58%. For 80 patients previously treated with crizotinib, the response rate was 56%. Responses were observed in patients with various resistance mutations in ALK, including L1196M, and in patients without detectable mutations. The median progression-free survival of patients receiving at least 400 mg of ceritinib was 7 months. Based on these findings, Ceritinib has recently been granted FDA approval for treatment of patients with ALK+ NSCLC in the second-line setting following failure or intolerance to crizotinib.

Multiple trials are nevertheless ongoing for this drug. These include three phase II trials of ceritinib for crizotinib-resistant patients (NCT01685060; NCT02040870) and crizotinib-naïve patients (NCT01685138). One phase II study is looking specifically at patients with ROS1 mutation (NCT01964157). Two phase III trials are comparing ceritinib with standard chemotherapy in patients previously treated with platinum-based chemotherapy (NCT01828112), and chemotherapy-naïve patients (NCT01828099). One phase I study is assessing the combination of ceritinib with a HSP90 inhibitor (NCT01772797).

### Alectinib (CH5424802/RO5424802)

Alectinib (Chugai and Roche Pharmaceuticals) is a highly potent selective ALK inhibitor with activity against L1196M gatekeeper mutation as well as other secondary mutations such as F1174L and R1275Q (34, 35). Results from a recent phase I/II study with alectinib in a Japanese population have been published (36). In the phase I study, alectinib was given up to a maximum dose of 300 mg twice daily without dose-limiting toxicity. In the phase II part of the study, 40/46 (87%) of patients achieved a partial response within 6 weeks of treatment. Two patients had a complete response. Median PFS had not been reached at the time of the report. Responses were seen in brain metastases in three patients. Grade 3 adverse events including neutropenia and increase creatine phosphokinase occurred in 12 (26%) of patients.

Three phase II/III trials with alectinib are in progress in crizotinib-naïve (ALEXA trial-NCT02075840) as well as in crizotinib-resistant (NCT01871805; NCT01801111) patients.

#### AP26113

AP26113 (Ariad Pharmaceuticals) is a novel inhibitor of ALK with activity against L1196M gatekeeper mutation as well as against ROS1 and EGFR (including mutant form with the T790M gatekeeper mutation) (37). In an ongoing phase I/II study (NCT01449461), the established dose was at 180 mg once daily with good antitumor activity in ALK+ NSLC patients (38). Objective response was observed in 15/24 (63%) patients (1 complete response and 14 partial responses), including 12/16 (75%) in patients resistant to crizotinib. Of interest, 4/5 patients with brain metastasis had objective responses as well. The most common treatment-related adverse events were nausea (33%), fatigue (22%), and diarrhea (20%). A confirmatory phase II studies in crizotinib-resistant (NCT02094573) is currently in progress.

### Other new-generation ALK agents

Multiple other new-generation ALK agents are currently under phase I study. These include TSR-001, ASP3026, PF-06463922, as well as X-396 (39–42). Details about these studies can be found in Table 1.

## HSP90 Inhibitors

Pre-clinical studies involving HSP90 inhibitors in ALK+ NSCLC led to decreased ALK fusion protein levels *in vitro*, and led to tumor regression in *in vivo* models (43). Currently, multiple HSP90 inhibitors are in phase I/II clinical trials involving ALK+ NSCLC patients, either as stand-alone drugs or in combination with other ALK-inhibitors such as crizotinib. Details about ongoing studies can be found in Table 1.

Early results from phase II studies involving IPI-504 (Infinity Pharma) in three NSCLC patients resulted in two partial responses and one case of prolonged stable disease (44). In another phase II study of ganetespib (STA-9090), 4/8 patients had partial responses and three patients had stable disease (45). The most common adverse effects were diarrhea, fatigue, nausea, and anorexia. Two patients in this study died of cardiac arrest and renal failure associated with ganetespib. There has also been one case report of a crizotinib-resistant ALK+ NSCLC with an objective response to this drug (31). AUY922 (Novartis) has been studied as a single agent in both crizotinib-resistant and – naïve patients. Objective response was achieved in 6/21 (29%) of patients, 4 of which were crizotinib-naïve and remainder 2 had been previously treated with crizotinib (46).

## Summary and Conclusion

The identification of the ALK fusion protein in 2007 and the fast development and approval of a FDA targeted treatment in <5 years constitutes a remarkable feat in the field of targeted therapies. Crizotinib holds many promises from its early debut in treatment-refractory NSCLC patients. Final results from phase III randomized trials using Crizotinib as a first-line therapy are eagerly awaited for. Despite excellent response in the initial stages, most patients develop resistance to crizotinib. Elucidating resistance mechanism and subsequently developing therapeutic strategies to overcome resistance to ALK-inhibitors constitutes a priority, with the vast majority of ongoing clinical trials in this field involving new-generation ALK-inhibitors or combination therapy with other targeted agents. Key questions remain on how to correctly identify the resistance mechanism of a tumor progressing on ALK-targeted therapy given re-biopsy is often technically challenging and resource intensive, as well as on how to correctly identify and stream the correct combination of therapies to the appropriate patient populations as first-line therapy. The ongoing Q-CROC-05 multicenter phase IV clinical trial (NCT02041468) will aim to shed some light on these important clinical issues.

## Conflict of Interest Statement

The authors declare that the research was conducted in the absence of any commercial or financial relationships that could be construed as a potential conflict of interest.

## Supplementary Material

The Supplementary Material for this article can be found online at http://www.frontiersin.org/Journal/10.3389/fonc.2014.00174/abstract
