# Supplementary material for: A Systemic Review of Resistance Mechanisms and Ongoing Clinical Trials in ALK-Rearranged Non-Small Cell Lung Cancer
Source: Front Oncol. 2014 Jul 21;4:174. doi: 10.3389/fonc.2014.00174 (PMC4104550; doi:10.3389/fonc.2014.00174)
Supplement: Supplementary file 1 [file Data_Sheet1.PDF]

Table S1 | Ongoing clinical trials with crizotinib.

Study	Phase	Number of patients	Primary objectives
PROFILE 1014 (NCT01154140)	Phase III	334	PFS versus pemetrexed plus cisplatin or carboplatin
East-Asian Study (NCT01639001)	Phase III	200	PFS versus pemetrexed plus cisplatin or carboplatin
PROFILE 1005 (NCT00932451)	Phase II	1100	ORR and adverse events and laboratory test abnormalities related to crizotinib
STUDY 1002 (NCT00965731)	Phase I/II	175	MTD of crizotinib plus erlotinib. PFS single agent versus combination therapy

PFS, progression-free survival; ORR, overall response rate; MTD, maximum tolerated dose.
